# Association of life’s simple 7 with osteoarthritis risk: a cross-sectional study from NHANES 2009–2018

**DOI:** 10.3389/fmed.2025.1511270

**Published:** 2025-04-09

**Authors:** Teng Ma, Zhiping Yu, Wenjing Qu, Xiaogeng Sun, Jian Huang, Wenpeng Xie, Haibo Cong

**Affiliations:** ^1^First Clinical Medical College, Shandong University of Traditional Chinese Medicine, Jinan, China; ^2^Department of Orthopedics, Limin Hospital of Weihai High District, Weihai, China; ^3^Department of Orthopedics, Affiliated Hospital of Shandong University of Traditional Chinese Medicine, Jinan, China; ^4^Medical Technology Innovation Center, Shandong First Medical University, Jinan, China

**Keywords:** osteoarthritis, Life’s Simple 7, NHANES, cardiovascular health, cross-sectional studies

## Abstract

**Background:**

The Life’s Simple 7 (LS7) metric is a comprehensive index evaluating cardiovascular health from a holistic perspective, integrating seven cardiovascular-related health factors and behaviors. However, the relationship between LS7 and the likelihood of developing osteoarthritis (OA) remains unclear. Therefore, this study investigated the possible association between LS7 and OA.

**Methods:**

Using data from the National Health and Nutrition Examination Survey from 2009 to 2018, 19,603 participants were included in this study. LS7 was treated as the independent variable, whereas OA served as the dependent variable. The association between LS7 and OA was assessed by performing a weighted logistic regression analysis. To evaluate the stability of the findings, subgroup analyses were conducted. Additionally, restricted cubic spline (RCS) analysis was conducted to explore potential nonlinear relationships between LS7 and OA.

**Results:**

LS7 scores were significantly negatively correlated with OA risk (OR: 0.808, 95% CI: 0.786–0.830). Analysis of LS7 categories indicated that individuals with ideal scores had a 55.9% lower OA risk than those with poor scores (OR: 0.559, 95% CI: 0.379–0.823). Subgroup analysis demonstrated that factors such as age, gender, marital status, BMI, and blood pressure moderated the relationship between LS7 and OA in inconsistent ways. The RCS analysis revealed a significant nonlinear negative association between LS7 scores and OA risk (*p*-nonlinear < 0.001).

**Conclusion:**

This study suggests a nonlinear negative correlation between LS7 and OA risk, implying that better cardiovascular health may be linked to a reduced risk of developing OA. However, the relationship varies across different subgroups.

## 1 Introduction

Osteoarthritis (OA) is a chronic joint disease primarily associated with aging and joint injury, characterized by joint pain and functional impairment (1, 2). OA frequently results in physical disability, significantly affecting the quality of life (3). In recent global disease assessments, OA has been recognized as a major risk factor for disability and mortality in older adults people, alongside cardiovascular disease (CVD), stroke, and chronic obstructive pulmonary disease (COPD) (4). As society rapidly ages, the rising prevalence of OA, particularly in the knee and hip, has emerged as a pressing public health issue that requires urgent attention (5).

Given that OA and CVD affect similar populations and share high disability rates, exploring the link between these two conditions has become a topic of significant research interest (6). Studies have suggested that OA and CVD share several common risk factors, such as obesity and inflammation (7). Moreover, OA has been identified as a significant risk factor for CVD development in the future (8). While cardiovascular-related factors, such as smoking, physical activity levels, and body mass index (BMI), play a crucial role in determining OA risk (9–11), the connection between cardiovascular health indicators and OA remains underexplored. This underscores the need for further research to clarify the effects of CVD prevention on OA development.

The American Heart Association (AHA) developed Life’s Simple 7 (LS7), a comprehensive measure of cardiovascular health that integrates key cardiovascular factors and behaviors to provide a holistic assessment of overall health (12). Individuals are scored based on their smoking status, physical activity level, BMI, total cholesterol, fasting glucose, blood pressure, and diet according to the LS7 criteria. These scores categorize individuals into poor, intermediate, and ideal cardiovascular health levels (13). Given the lack of research exploring the impact of cardiovascular health on the risk of developing OA, LS7 scores are ideally suited to represent levels of cardiovascular health for this study. Numerous studies have demonstrated significant correlations between LS7 scores and the risk of various diseases beyond cardiovascular health (14). However, to date, no study has specifically applied LS7 scores to investigate potential associations between cardiovascular health and OA.

In this context, we explored the relationship between LS7 scores and OA. This research constitutes a cross-sectional analysis using information gathered from the National Health and Nutrition Examination Survey (NHANES). We hypothesized that the higher the LS7 scores, the lower the risk of developing OA. Therefore, LS7 scores were considered as the independent variable and having OA as the dependent variable. The association between LS7 scores and the risk of OA was explored by weighted logistic regression analysis, subgroup analysis, and RCS analysis.

## 2 Materials and methods

### 2.1 Data sources and study population

The National Center for Health Statistics (NCHS) conducts the NHANES,^1^ a national, ongoing cross-sectional survey that employs a stratified, multistage probability design. The population of the entire country is meticulously divided into strata based on demographic and geographic characteristics. Within each stratum, discrete clusters are identified and families are then randomly selected for recruitment. The survey collects a wide range of data, including demographic, socioeconomic, dietary, and laboratory information, through interviews and physical examinations. The NCHS Institutional Review Committee has approved this program, and all participants provide informed consent.

**FIGURE 1:**
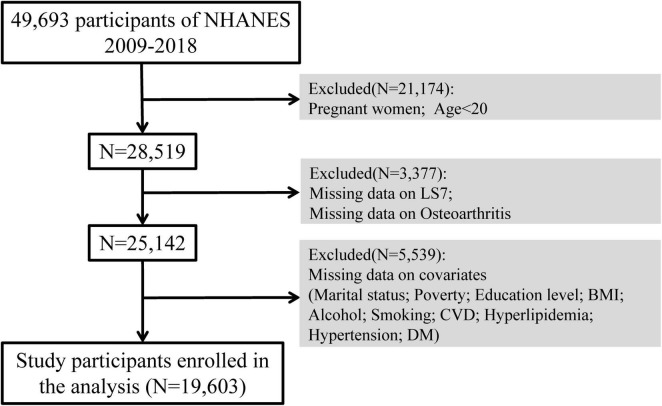
Flowchart of participant selection.

For this study, we used NHANES data from 2009 to 2018. Across five survey cycles, 49,693 participants were included. After excluding pregnant participants, individuals below the age of 20 (*N* = 21,174), with those missing LS7 scores or OA data (*N* = 3,377), and participants with missing covariate data (*N* = 5,539), the final analysis included 19,603 participants (Figure 1).

### 2.2 Measurement of LS7 scores

The LS7 questionnaire assesses three health-related behaviors (dietary patterns, physical activity levels, and tobacco use) along with four health metrics: BMI, blood pressure readings, blood glucose levels, and total cholesterol (12). Dietary habits were measured using the Healthy Eating Index (HEI-2015) scores (15), derived from two 24-h dietary recall interviews in which participants provided detailed accounts of their food consumption. The physical activity of the participants was assessed using self-reported questionnaires, which captured the frequency and duration of moderate or vigorous physical activities, such as walking, jogging, running, bicycling, or swimming, over the past 30 days. Smoking behavior was similarly evaluated using self-reported questionnaires. During the physical examination, participants’ height and weight were recorded, and BMI was calculated as weight in kilograms divided by height in meters squared (kg/m^2^). According to the criteria established by the National Institutes of Health, BMI was categorized as underweight (< 18.5 kg/m^2^), normal (18.5–24.9 kg/m^2^), overweight (25.0–29.9 kg/m^2^), and obese (≥ 30 kg/m^2^). Blood pressure was determined by averaging three consecutive measurements taken during the physical exam. Blood glucose and total cholesterol levels were obtained from participants’ serum total cholesterol and glycosylated hemoglobin values. Based on prior studies, the overall LS7 score was categorized into three levels: poor (0–7), intermediate (8–10), and ideal (11–14) (16).

### 2.3 Assessment of OA

Participants were screened for OA using a questionnaire that asked if a doctor had ever diagnosed them with arthritis. Notably, self-reported arthritis is commonly used as a case definition in epidemiologic studies (17). One study showed an 81% concordance between self-reported and clinically confirmed diagnoses for OA, indicating that self-reported OA is generally reliable (18). Those who answered yes were directed to further questionnaires where they specified the type of arthritis. Participants who selected OA were included in the study.

### 2.4 Covariates

Several potential confounding factors were considered in the analysis, such as age, sex, ethnicity, relationship status, educational attainment, poverty-to-income ratio (PIR), alcohol consumption, cardiovascular disease (CVD), high cholesterol levels, high blood pressure, and diabetes mellitus (DM). Age was categorized into two groups: < 60 years and ≥ 60 years, taking into account cardiovascular risk factors and the age of the population susceptible to OA. Ethnicity options included non-Hispanic white, Mexican American, non-Hispanic black or others. Marital status options included married, living with partner, widowed, never married, divorced or separated. Education levels were categorized as less than high school, high school or more than high school. PIR ≤ 1 was considered to be below the federal poverty level and therefore categorized as ≤ 1 or > 1. Alcohol consumption status was defined as never (less than 12 drinks in a lifetime), ever (≥ 12 drinks in 1 year and no drinks in the last year, or no drinks in the last year but ≥ 12 drinks in a lifetime), mild, moderate, or heavy. History of CVD was determined by self-report of prior diagnoses, including heart failure, coronary artery disease, angina, and so on. Hyperlipidemia, hypertension and DM were all categorized as either yes (abnormal objective indicators, doctor-confirmed diagnosis, or taking related medications) or no.

### 2.5 Statistical analysis

In the descriptive analysis, continuous variables were represented using the mean and standard deviation (SD), whereas categorical variables were displayed as frequency and weighted percentage. For categorical data, the Chi-square test was applied, and to compare group differences in continuous variables, the *t*-test was utilized. We employed survey-weighted logistic regression to assess the relationship between LS7 metrics and OA. Three models were developed for the multivariate test. Model 1 accounted for age, gender, and race. Model 2 included adjustments for age, gender, race, marital status, PIR, education level, BMI, alcohol consumption, and smoking status. Model 3 incorporated all covariates. Additionally, subgroup analyses were conducted to determine if these associations differed across various groups. RCS were used within weighted logistic regression models to explore the nonlinear relationships between the continuous LS7 scores and OA.

## 3 Results

### 3.1 Baseline characteristics of participants

Data from 19,603 participants were incorporated in the analysis. Table 1 presents the baseline characteristics of the study population, categorized by OA risk. A higher percentage of people with OA were aged ≥ 60 years than the non-OA population (non-OA vs. OA 19.4 vs. 59.75, *p* < 0.001). A higher proportion of OA patients were female (non-OA vs. OA 48.23 vs. 64.71, *p* < 0.001). Non-Hispanic whites comprised a larger percentage of the OA population compared with the non-OA population (non-OA vs. OA 65.51 vs. 83.19, *p* < 0.001). The percentage of married individuals was higher in the OA population (61.43%) than in the non-OA population (53.69%), whereas unmarried individuals were less common in the OA group (6.86%) compared with the non-OA group (21.18%). A greater proportion of the OA population had a PIR > 1 than the non-OA population (non-OA vs. OA 85.1 vs. 89.8, *p* < 0.001). Nearly half of the OA group had a BMI ≥ 30 (49.58%) compared with only 36.26% in the non-OA group. People with OA were more likely to be light drinkers, former smokers, and have CVD, hyperlipidemia, hypertension, and DM compared with those without OA. In addition, patients with OA had significantly lower LS7 scores than those without OA (non-OA vs. OA 8.5 vs. 7.21, *p* < 0.001). These results suggest that factors such as age, gender, race, marital status, PIR, BMI, alcohol consumption, smoking status, CVD, hyperlipidemia, hypertension, DM, and LS7 scores are potentially associated with an increased OA risk.

**TABLE 1:** The characteristics of participants.

Variables	Total (weighted %)	No-OA (weighted %)	OA (weighted %)	*P*-value (weighted %)
**Age**				< 0.001
< 60	13,620 (75.18)	12,794 (80.60)	826 (40.25)	
≥ 60	5,983 (24.82)	4,448 (19.40)	1,535 (59.75)	
**Sex**				< 0.001
Male	9,815 (49.56)	8,964 (51.77)	851 (35.29)	
Female	9,788 (50.44)	8,278 (48.23)	1,510 (64.71)	
**Race**				< 0.001
Non-Hispanic white	8,206 (67.88)	6,760 (65.51)	1,446 (83.19)	
Mexican American	2,756 (8.17)	2,564 (8.98)	192 (2.97)	
Non-Hispanic black	4,046 (10.43)	3,672 (11.06)	374 (6.33)	
Others	4,595 (13.52)	4,246 (14.46)	349 (7.51)	
**Marital status**				< 0.001
Married	9,966 (54.73)	8,679 (53.69)	1,287 (61.43)	
Living with partner	1,698 (8.48)	1,615 (9.28)	83 (3.33)	
Widowed	1,320 (5.04)	938 (3.78)	382 (13.14)	
Never married	3,868 (19.26)	3,687 (21.18)	181 (6.86)	
Divorced	2,138 (10.30)	1,769 (9.80)	369 (13.56)	
Separated	613 (2.18)	554 (2.26)	59 (1.68)	
**Education level**				0.05
High school	4,344 (22.25)	3,833 (22.43)	511 (21.10)	
Less than High school	4,061 (13.24)	3,620 (13.48)	441 (11.72)	
More than High school	11,198 (64.51)	9,789 (64.09)	1,409 (67.18)	
**PIR**				< 0.001
≤ 1	4,178 (14.27)	3,777 (14.90)	401 (10.20)	
> 1	15,425 (85.73)	13,465 (85.10)	1,960 (89.80)	
**BMI**				< 0.001
≥ 25 to < 30	6,348 (32.38)	5,660 (32.68)	688 (30.41)	
< 25	5,738 (29.57)	5,266 (31.06)	472 (20.01)	
≥ 30	7,517 (38.05)	6,316 (36.26)	1,201 (49.58)	
**Alcohol**				< 0.001
Never	2,710 (10.34)	2,400 (10.45)	310 (9.65)	
Former	2,740 (11.28)	2,266 (10.49)	474 (16.40)	
Mild	6,844 (37.62)	5,883 (36.54)	961 (44.59)	
Moderate	3,166 (18.53)	2,813 (18.81)	353 (16.75)	
Heavy	4,143 (22.23)	3,880 (23.72)	263 (12.62)	
**Smoking**				< 0.001
Never	11,113 (56.88)	9,984 (58.11)	1,129 (48.93)	
Former	4,529 (24.25)	3,704 (22.53)	825 (35.35)	
Now	3,961 (18.87)	3,554 (19.36)	407 (15.72)	
**CVD**				< 0.001
No	17,678 (92.02)	15,829 (93.59)	1,849 (81.88)	
Yes	1,925 (7.98)	1,413 (6.41)	512 (18.12)	
**Hyperlipidemia**				< 0.001
No	6,338 (32.93)	5,895 (35.13)	443 (18.71)	
Yes	13,265 (67.07)	11,347 (64.87)	1,918 (81.29)	
**Hypertension**				< 0.001
No	11,773 (64.18)	10,978 (68.15)	795 (38.60)	
Yes	7,830 (35.82)	6,264 (31.85)	1,566 (61.40)	
**DM**				< 0.001
No	16,074 (86.21)	14,411 (87.83)	1,663 (75.73)	
Yes	3,529 (13.79)	2,831 (12.17)	698 (24.27)	
**LS7 group**				< 0.001
Poor	8,163 (36.05)	6,724 (33.22)	1,439 (54.30)	
Intermediate	8,070 (44.06)	7,272 (44.85)	798 (39.01)	
Ideal	3,370 (19.89)	3,246 (21.93)	124 (6.69)	
LS7	8.33 (0.04)	8.50 (0.04)	7.21 (0.08)	< 0.001

### 3.2 Association between LS7 and OA

Table 2 shows that the odds ratio (OR) for LS7 was 0.808 [95% confidence interval (CI): 0.786–0.830], indicating that LS7 was significantly negatively correlated with the likelihood of developing OA (*p* < 0.001). In Model 2, after adjustments were made for age, gender, and race, the negative correlation remained statistically significant (OR = 0.853, 95% CI: 0.828–0.879, *p* < 0.001). Even after all covariates were controlled in Model 4, the negative association remained significant. Specifically, for each 1-unit increase in LS7 score, the OA risk was reduced by 5.2% (OR = 0.948, 95% CI: 0.906–0.991, *p* = 0.02). When LS7 scores were analyzed as categorical variables, participants with optimal LS7 scores had a lower OA risk. Notably, individuals in the ideal-scoring group had a substantially reduced OR for the OA risk compared with those in the poor-scoring group. After adjustments were made for all covariates, the ideal group had a 55.9% decreased likelihood of developing OA compared with the poor group (OR = 0.559, 95% CI: 0.379–0.823, *p* = 0.004).

**TABLE 2:** Association between LS7 and OA.

	Model 1		Model 2		Model 3		Model 4	
**Character**	**OR (95% CI)**	***P*-value**	**OR (95% CI)**	***P*-value**	**OR (95% CI)**	***P*-value**	**OR (95% CI)**	***P*-value**
LS7	0.808 (0.786, 0.830)	< 0.001	0.853 (0.828, 0.879)	< 0.001	0.893 (0.856, 0.931)	< 0.001	0.948 (0.906, 0.991)	0.020
**LS7–Group**								
Poor	Ref		Ref		Ref		Ref	
Intermediate	0.532 (0.452, 0.626)	< 0.001	0.656 (0.558, 0.772)	< 0.001	0.779 (0.651, 0.931)	0.007	0.927 (0.770, 1.115)	0.413
Ideal	0.187 (0.142, 0.246)	< 0.001	0.277 (0.205, 0.375)	< 0.001	0.399 (0.275, 0.579)	< 0.001	0.559 (0.379, 0.823)	0.004
*P* for trend		< 0.001		< 0.001		< 0.001		0.016

Model 1: no covariates were adjusted. Model 2: Age, sex, race. Model 3: Age, sex, race, marital status, PIR, education level, BMI, alcohol, smoking. Model 4: Age, sex, race, marital status, PIR, education level, BMI, alcohol, smoking, CVD, hypertension, hyperlipidemia, DM.

### 3.3 Subgroup analyses

**FIGURE 2:**
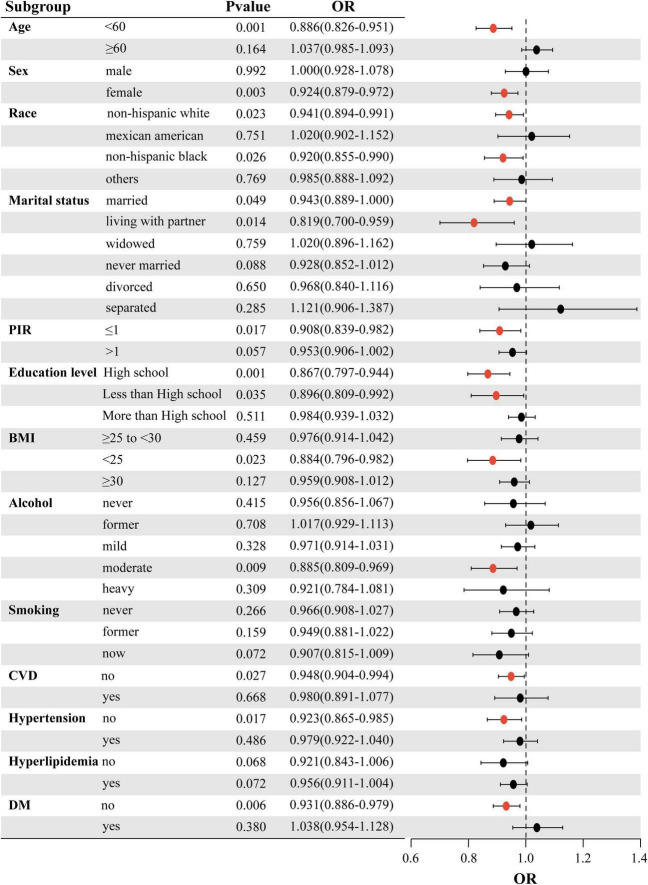
Subgroup analysis of the association between LS7 and OA.

To further evaluate the strength of the association between LS7 scores and the likelihood of developing OA, subgroup analyses were performed based on various factors such as age, sex, race, marital status, PIR, educational attainment, BMI, alcohol consumption, smoking habits, CVD, blood pressure, lipid levels, and blood glucose. The results of these analyses are illustrated in a forest plot (Figure 2). The analysis confirmed that LS7 scores were inversely related to the risk of developing OA (Table 3). Notably, the effects of demographic factors such as age, gender, race, marital status, education, BMI, alcohol consumption, smoking, CVD, blood pressure, and blood glucose on the association between LS7 scores and the OA risk were inconsistent across groups. Key demographic characteristics associated with the likelihood of developing OA were age < 60 years (OR = 0.886, 95% CI: 0.826–0.951), female gender (OR = 0.924, 95% CI: 0.879–0.972), non-Hispanic white race (OR = 0.941, 95% CI: 0.894–0.991), living with a partner

**TABLE 3:** Subgroup analysis of the relation between the LS7 and OA.

Character	OR (95% CI)	*P*-value	*P* for interaction
**Age**			< 0.001
< 60	0.886 (0.826, 0.951)	0.001	
≥ 60	1.037 (0.985, 1.093)	0.164	
**Sex**			0.008
Male	1.000 (0.928, 1.078)	0.992	
Female	0.924 (0.879, 0.972)	0.003	
**Race**			0.092
Non-Hispanic white	0.941 (0.894, 0.991)	0.023	
Mexican American	1.020 (0.902, 1.152)	0.751	
Non-Hispanic black	0.920 (0.855, 0.990)	0.026	
Others	0.985 (0.888, 1.092)	0.769	
**Marital status**			0.013
Married	0.943 (0.889, 1.000)	0.049	
Living with partner	0.819 (0.700, 0.959)	0.014	
Widowed	1.020 (0.896, 1.162)	0.759	
Never married	0.928 (0.852, 1.012)	0.088	
Divorced	0.968 (0.840, 1.116)	0.650	
Separated	1.121 (0.906, 1.387)	0.285	
**PIR**			0.075
≤ 1	0.908 (0.839, 0.982)	0.017	
> 1	0.953 (0.906, 1.002)	0.057	
**Education level**			0.352
High school	0.867 (0.797, 0.944)	0.001	
Less than High school	0.896 (0.809, 0.992)	0.035	
More than High school	0.984 (0.939, 1.032)	0.511	
**BMI**			0.012
≥ 25 to < 30	0.976 (0.914, 1.042)	0.459	
< 25	0.884 (0.796, 0.982)	0.023	
≥ 30	0.959 (0.908, 1.012)	0.127	
**Alcohol**			0.15
Never	0.956 (0.856, 1.067)	0.415	
Former	1.017 (0.929, 1.113)	0.708	
Mild	0.971 (0.914, 1.031)	0.328	
Moderate	0.885 (0.809, 0.969)	0.009	
Heavy	0.921 (0.784, 1.081)	0.309	
**Smoking**			0.135
Never	0.966 (0.908, 1.027)	0.266	
Former	0.949 (0.881, 1.022)	0.159	
Now	0.907 (0.815, 1.009)	0.072	
**CVD**			0.065
No	0.948 (0.904, 0.994)	0.027	
Yes	0.980 (0.891, 1.077)	0.668	
**Hypertension**			0.003
No	0.923 (0.865, 0.985)	0.017	
Yes	0.979 (0.922, 1.040)	0.486	
**Hyperlipidemia**			0.077
No	0.921 (0.843, 1.006)	0.068	
Yes	0.956 (0.911, 1.004)	0.072	
**DM**			0.102
No	0.931 (0.886, 0.979)	0.006	
Yes	1.038 (0.954, 1.128)	0.380	

(OR = 0.819, 95% CI: 0.700–0.959), PIR ≤ 1 (OR = 0.908, 95% CI: 0.839–0.982), high school education (OR = 0.867, 95% CI: 0.797–0.944), BMI < 25 (OR = 0.884, 95% CI: 0.796–0.982), moderate alcohol consumption (OR = 0.885, 95% CI: 0.809–0.969), no CVD (OR = 0.948, 95% CI: 0.904–0.994), no hypertension (OR = 0.923, 95% CI: 0.865–0.985), and no DM (OR = 0.931, 95% CI: 0.886–0.979).

When analyzing the group with low LS7 scores in relation to the one with high scores, a similar negative correlation between LS7 and risk of developing OA was observed (Table 4). However, the effects of age, gender, race, marriage, educational background, BMI, alcohol consumption, smoking habits, blood pressure, and blood glucose levels on the connection between LS7 scores and the likelihood of developing OA varied among different groups.

**TABLE 4:** Subgroup analysis of the relation between the LS7 classification and OA.

Character	Poor	Intermediate	*P*-value	Ideal	*P*-value	*P* for trend	*P* for interaction
**Age**							< 0.001
< 60	Ref	0.868 (0.646, 1.167)	0.342	0.400 (0.230, 0.696)	0.002	0.006	
≥ 60	Ref	1.011 (0.824, 1.241)	0.911	1.051 (0.633, 1.746)	0.845	0.853	
**Sex**							0.026
Male	Ref	1.129 (0.863, 1.476)	0.370	0.860 (0.482, 1.534)	0.602	0.937	
Female	Ref	0.822 (0.658, 1.027)	0.083	0.455 (0.289, 0.716)	0.001	0.002	
**Race**							0.087
Non-Hispanic white	Ref	0.941 (0.752, 1.178)	0.590	0.568 (0.363, 0.889)	0.014	0.05	
Mexican American	Ref	0.816 (0.506, 1.316)	0.395	0.672 (0.276, 1.638)	0.373	0.256	
Non-Hispanic black	Ref	0.696 (0.504, 0.961)	0.029	0.421 (0.203, 0.875)	0.021	0.006	
Others	Ref	1.005 (0.665, 1.518)	0.982	0.496 (0.241, 1.024)	0.058	0.218	
**Marital status**							0.029
Married	Ref	0.950 (0.756, 1.193)	0.654	0.644 (0.412, 1.007)	0.054	0.105	
Living with partner	Ref	0.604 (0.317, 1.153)	0.124	0.095 (0.018, 0.514)	0.007	0.006	
Widowed	Ref	0.865 (0.553, 1.351)	0.516	0.960 (0.299, 3.076)	0.944	0.642	
Never married	Ref	1.022 (0.573, 1.825)	0.939	0.334 (0.137, 0.814)	0.017	0.044	
Divorced	Ref	1.008 (0.600, 1.693)	0.977	0.604 (0.228, 1.600)	0.304	0.539	
Separated	Ref	0.749 (0.254, 2.205)	0.593	0.000 (0.000, 0.000)	< 0.001	0.214	
**PIR**							0.039
≤ 1	Ref	0.646 (0.433, 0.963)	0.033	0.241 (0.106, 0.545)	< 0.001	0.001	
> 1	Ref	0.958 (0.786, 1.166)	0.661	0.596 (0.395, 0.899)	0.015	0.05	
**Education level**							0.04
High school	Ref	0.640 (0.468, 0.874)	0.006	0.521 (0.255, 1.065)	0.073	0.007	
Less than High school	Ref	0.699 (0.478, 1.021)	0.063	0.743 (0.283, 1.948)	0.539	0.105	
More than High school	Ref	1.064 (0.859, 1.317)	0.566	0.617 (0.393, 0.967)	0.036	0.147	
**BMI**							0.343
≥ 25 to < 30	Ref	0.996 (0.759, 1.308)	0.978	0.488 (0.274, 0.868)	0.016	0.06	
< 25	Ref	0.987 (0.676, 1.443)	0.947	0.613 (0.331, 1.134)	0.117	0.092	
≥ 30	Ref	0.802 (0.620, 1.038)	0.092	0.962 (0.246, 3.764)	0.955	0.198	
**Alcohol**							0.571
Never	Ref	0.877 (0.594, 1.295)	0.504	0.846 (0.319, 2.241)	0.732	0.608	
Former	Ref	1.269 (0.928, 1.735)	0.132	1.041 (0.437, 2.478)	0.926	0.351	
Mild	Ref	0.922 (0.722, 1.177)	0.509	0.543 (0.326, 0.905)	0.020	0.043	
Moderate	Ref	0.863 (0.589, 1.266)	0.445	0.308 (0.132, 0.720)	0.007	0.012	
Heavy	Ref	0.805 (0.433, 1.498)	0.487	0.591 (0.150, 2.328)	0.446	0.414	
**Smoking**							0.924
Never	Ref	0.976 (0.769, 1.239)	0.839	0.741 (0.446, 1.232)	0.242	0.318	
Former	Ref	0.865 (0.657, 1.139)	0.296	0.411 (0.214, 0.791)	0.009	0.032	
Now	Ref	0.783 (0.492, 1.246)	0.296	0.349 (0.090, 1.355)	0.126	0.165	
**CVD**							0.701
No	Ref	0.946 (0.776, 1.152)	0.571	0.603 (0.397, 0.916)	0.019	0.048	
Yes	Ref	0.895 (0.635, 1.260)	0.517	0.287 (0.084, 0.979)	0.046	0.124	
**Hypertension**							0.621
No	Ref	0.971 (0.739, 1.274)	0.827	0.615 (0.377, 1.004)	0.052	0.072	
Yes	Ref	0.924 (0.727, 1.173)	0.508	0.571 (0.288, 1.131)	0.106	0.261	
**Hyperlipidemia**							0.418
No	Ref	0.953 (0.637, 1.427)	0.813	0.501 (0.258, 0.974)	0.042	0.054	
Yes	Ref	0.912 (0.750, 1.109)	0.349	0.625 (0.405, 0.964)	0.034	0.06	
**DM**							0.38
No	Ref	0.886 (0.724, 1.083)	0.232	0.538 (0.359, 0.806)	0.003	0.007	
Yes	Ref	1.185 (0.858, 1.635)	0.296	0.702 (0.168, 2.938)	0.622	0.356	

### 3.4 RCS analysis

**FIGURE 3:**
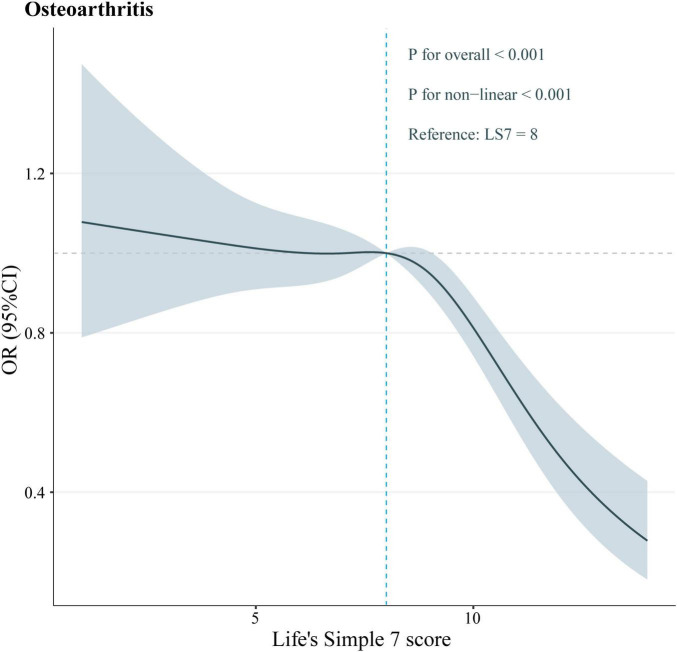
The RCS plot between LS7 score and OA.

To determine if a nonlinear relationship existed between LS7 scores and the likelihood of developing OA, the data were analyzed using the RCS test. The analysis unveiled that LS7 scores and OA were significantly and nonlinearly associated (*p* nonlinear < 0.05, Figure 3). Specifically, the negative effect of LS7 scores on the OA risk was more pronounced when the scores were higher than 8.

## 4 Discussion

This study is the first to investigate the connection between the LS7 metrics of the American Heart Association and OA. The main conclusion is that LS7 scores are nonlinearly and negatively associated with the OA risk. This finding suggests that stricter adherence to these cardiovascular health indicators correlates with a lower OA risk. Notably, the OA risk is significantly reduced when LS7 scores exceed the “intermediate” level. This connection continues to be important even when accounting for possible confounding factors. Findings from this study indicate that it is essential to emphasize compliance with LS7 metrics among the general populace, as this may not only lower the chances of CVD but also help decrease the likelihood of OA development.

Notably, the higher CVD risk in OA patients complicates OA treatment. Specifically, OA patients undergoing surgery face a greater risk for intraoperative and postoperative complications, including anesthetic complications, venous thromboembolism, and myocardial infarction (19). Furthermore, OA patients who also have CVD are at a significantly increased risk when oral nonsteroidal anti-inflammatory medications are used (20). Therefore, exploring the relationship and common pathogenesis between OA and CVD is crucial for improving OA treatment. Studies have indicated that individuals with OA are over two times more likely to experience heart failure or ischemic heart disease than those without OA (21). This highlights the close link between OA and CVD. Some studies have suggested a coordinated relationship between OA and CVD in older adults (22). Both OA and atherosclerosis are processes characterized by inflammation and the involvement of inflammatory mediators. Inflammatory responses are present in vessel walls, joints, and synovium of both OA and CVD patients (23, 24). High levels of pro-inflammatory factors are a major cause of chondrocyte apoptosis and abnormal cartilage metabolism (25). Additionally, the inflammatory process contributes to vascular inflammation and atherosclerosis, ultimately leading to CVD development (26).

We observed a clinically significant association between LS7 scores and OA, particularly impacting musculoskeletal and cardiovascular health. Our results indicated that individuals with ideal LS7 scores had a significantly lower likelihood of developing OA by 44.1% compared with those with poor scores. Clinically, these results carry several important implications. Initially, they highlight the relationship between cardiovascular health and OA (21, 22, 27), reinforcing the idea of a shared pathophysiological foundation. Additionally, the finding that individuals with ideal LS7 scores exhibit a lower prevalence of OA suggests that efforts to enhance cardiovascular health could positively influence the prevention of OA. Moreover, recognizing adjustable risk factors within the LS7 framework provides clinicians with actionable goals for interventions aimed at reducing the likelihood of developing OA. Following and improving upon lifestyle measures outlined in LS7, including suitable levels of physical activity, quitting smoking, and maintaining a nutritious diet, can not only benefit cardiovascular well-being but also show significant potential for the prevention of OA.

Although the LS7 metrics primarily reflect cardiovascular health, the factors involved, such as BMI, physical activity status, and blood glucose levels, are closely linked to OA onset and progression. Numerous studies have indicated a clear connection between obesity, measured by BMI, and the incidence of OA (28, 29). Moreover, BMI is recognized as a predictor of knee pain, although it is not directly associated with radiographic features of OA (30). Customized physical interventions based on physical activity levels can significantly enhance health outcomes by improving pain, joint mobility, muscle nutrition, and balance (31). Importantly, no increased risk of OA has been found with low-, moderate-, or high-intensity exercise; rather, regular physical activity appears to prevent the development of OA risk factors such as obesity and muscle weakness (32). From a mechanistic standpoint, the adipomyokine Metrnl—highly expressed in skeletal muscle and released into circulation after exercise—has anti-inflammatory properties and inhibits chondrocyte pyroptosis, thus protecting articular cartilage (33). Furthermore, glucose plays a crucial role in maintaining anaerobic metabolism in particular chondrocytes; adequate glucose levels are believed to disrupt the inflammatory cycle in these cells, promoting cartilage repair (34). However, some clinical studies have reported that diabetic patients are at a higher OA risk than the general population, suggesting that hyperglycemic states predispose individuals to OA (35). The synovial inflammatory response tends to be more severe in OA patients with DM than in non-diabetic counterparts, probably due to the high-glycemic environment stimulating the accumulation of advanced glycosylation end products, endoplasmic reticulum stress, and inflammation in fibroblast-like synoviocytes. This inflammatory cascade can ultimately lead to chondrocyte degeneration (36).

The study adopted an inclusion criterion of age > 20 years, with subsequent stratification into ≥ 60 years and < 60 years. This design serves dual purposes: First, as OA is an age-related degenerative disease, clinically significant changes typically emerge in middle adulthood. Excluding individuals < 20 years minimizes confounding from adolescent skeletal development. Second, broad initial inclusion preserves sample diversity, enhancing result generalizability. However, merged inclusion may introduce intra-group heterogeneity. Thus, our subgroup analyses have the dual advantage of revealing the age-dependent relationship between LS7 scores and OA risk and effectively controlling for survivor bias through age stratification. However, a more detailed subgroup analysis would be more conducive to eliminating bias. Based on this, we plan that age continuity analysis will be performed in subsequent large-sample cohort studies.

Our subgroups analyses, revealed that certain participants’ characteristics influenced the correlation between LS7 scores and OA. For example, increasing LS7 scores significantly reduced the OA risk in individuals aged < 60 years. This suggests that, as individuals age, LS7 scores become less effective in mitigating the effects of aging on cartilage degeneration. Furthermore, adherence to LS7 criteria was particularly beneficial in preventing OA among female participants. Women are more susceptible to developing OA due to factors such as decreased muscle strength and higher body weight (37). Increased physical activity can enhance muscle strength and promote weight loss, subsequently reducing the OA risk (32). Interestingly, the LS7 score significantly negatively moderated the OA risk in individuals with BMI < 25. Because obesity is a major contributor to OA (38), and BMI is a key component of the LS7 metrics, the moderating effect of LS7 scores would be diminished in the absence of weight loss. Additionally, individuals without hypertension demonstrated a greater ability to lower their risk of OA by boosting their LS7 scores. Given that hypertension is a recognized risk factor for OA (39) and is included in the LS7 metrics, the protective effect of increasing LS7 scores on OA risk may be limited in patients with hypertension.

Notably, our RCS analyses unveiled a threshold effect of LS7 scores on the OA risk, with the turning point occurring at an LS7 score of 8. Below this critical threshold, the OA risk remained relatively stable. However, above this threshold, the negative impact of LS7 scores on the OA risk increased significantly. This finding suggests that strict adherence to the LS7 metrics and efforts to elevate LS7 scores above 8 may effectively reduce the OA risk.

We believe that our study possesses notable strengths. First, it used the NHANES database, which provided a sufficiently large and representative sample size, allowing for the first exploration of the relationship between the LS7 index and OA. Second, we adjusted for confounding factors to ensure more reliable findings. Furthermore, our study conducted subgroup analyses to assess the consistency of the relationship between LS7 scores and OA across different populations. However, some limitations should be acknowledged. As a cross-sectional study, we could not establish a causal link between LS7 scores and OA. While we accounted for various covariates, the influence of all potential confounding factors could not be eliminated. Additionally, certain key variables, such as OA, were determined through questionnaires, which may introduce recall bias into our findings.

## 5 Conclusion

Using the NHANES database, this research identified a potential nonlinear negative correlation between LS7 indicators and the OA risk. Adhering to the LS7 indicators not only supports cardiovascular health but also holds significant potential for OA prevention.

## Data Availability

The original contributions presented in this study are included in this article/supplementary material, further inquiries can be directed to the corresponding authors.

## Footnotes

1
https://www.cdc.gov/nchs/nhanes/index.htm

## References

[1] Tong L Yu H Huang X Shen J Xiao G Chen L Current understanding of osteoarthritis pathogenesis and relevant new approaches. *Bone Res.* (2022) 10:60. 10.1038/s41413-022-00226-9 36127328 PMC9489702

[2] Bannuru R Osani M Vaysbrot E Arden N Bennell K Bierma-Zeinstra S OARSI guidelines for the non-surgical management of knee, hip, and polyarticular osteoarthritis. *Osteoarthritis Cartilage.* (2019) 27:1578–89. 10.1016/j.joca.2019.06.011 31278997

[3] James S Abate D Abate K Abay S Abbafati C Abbasi N Global, regional, and national incidence, prevalence, and years lived with disability for 354 diseases and injuries for 195 countries and territories, 1990-2017: A systematic analysis for the Global Burden of Disease Study 2017. *Lancet.* (2018) 392:1789–858. 10.1016/S0140-6736(18)32279-7 30496104 PMC6227754

[4] Vos T Lim S Abbafati C Abbas K Abbasi M Abbasifard M Global burden of 369 diseases and injuries in 204 countries and territories, 1990-2019: A systematic analysis for the Global Burden of Disease Study 2019. *Lancet.* (2020) 396:1204–22. 10.1016/S0140-6736(20)30925-9 33069326 PMC7567026

[5] Long H Liu Q Yin H Wang K Diao N Zhang Y Prevalence trends of site-specific osteoarthritis from 1990 to 2019: Findings from the global burden of disease study 2019. *Arthritis Rheumatol.* (2022) 74:1172–83. 10.1002/art.42089 35233975 PMC9543105

[6] Perruccio A Zahid S Yip C Power J Canizares M Heckman G Cardiovascular risk profile and osteoarthritis-considering sex and multisite joint involvement: A Canadian longitudinal study on aging. *Arthritis Care Res.* (2022) 75:893–901. 10.1002/acr.24826 34825501

[7] Fernandes G Valdes A. Cardiovascular disease and osteoarthritis: Common pathways and patient outcomes. *Eur J Clin Invest.* (2015) 45:405–14. 10.1111/eci.12413 25630589

[8] Schieir O Hogg-Johnson S Glazier R Badley E. Sex variations in the effects of arthritis and activity limitation on first heart disease event occurrence in the Canadian general population: Results from the longitudinal national population health survey. *Arthritis Care Res.* (2016) 68:811–8. 10.1002/acr.22764 26473753

[9] Zhu S Ji L He Z Zhang W Tong Y Luo J Association of smoking and osteoarthritis in US (NHANES 1999-2018). *Sci Rep.* (2023) 13:3911. 10.1038/s41598-023-30644-6 36890196 PMC9995311

[10] Huang C Guo Z Feng Z Xu J Pan Z Liu W Comparative study on the association between types of physical activity, physical activity levels, and the incidence of osteoarthritis in adults: The NHANES 2007-2020. *Sci Rep.* (2024) 14:20574. 10.1038/s41598-024-71766-9 39232062 PMC11374984

[11] Ho J Mak C Sharma V To K Khan W. Mendelian randomization studies of lifestyle-related risk factors for osteoarthritis: A PRISMA review and meta-analysis. *Int J Mol Sci.* (2022) 23:11906. 10.3390/ijms231911906 36233208 PMC9570129

[12] Lloyd-Jones D Hong Y Labarthe D Mozaffarian D Appel L Van Horn L Defining and setting national goals for cardiovascular health promotion and disease reduction: The American Heart Association’s strategic Impact Goal through 2020 and beyond. *Circulation.* (2010) 121:586–613. 10.1161/CIRCULATIONAHA.109.192703 20089546

[13] Zou J Lin R Miao Y Xie M Wang X Gao L Association between Life’s simple 7 and post-stroke depression symptom from 2005-2016 NHANES survey: A cross-sectional study. *J Psychiatr Res.* (2024) 177(177):346–51. 10.1016/j.jpsychires.2024.07.005 39079467

[14] Wang L Chang G Cai S Zou X Qin M Tan Y. The association of Life’s Simple 7 and infertility among U.S. women. *Front Endocrinol.* (2024) 15:1288289. 10.3389/fendo.2024.1288289 38362273 PMC10867239

[15] Krebs-Smith S Pannucci T Subar A Kirkpatrick S Lerman J Tooze J Update of the healthy eating index: HEI-2015. *J Acad Nutr Diet.* (2018) 118:1591–602. 10.1016/j.jand.2018.05.021 30146071 PMC6719291

[16] Aneni E Crippa A Osondu C Valero-Elizondo J Younus A Nasir K Estimates of mortality benefit from ideal cardiovascular health metrics: A dose response meta-analysis. *J Am Heart Assoc.* (2017) 6:e006904. 10.1161/JAHA.117.006904 29269350 PMC5779012

[17] Kadier K Dilixiati D Zhang X Li H Kuang L Huang J Rheumatoid arthritis increases the risk of heart failure: Results from the cross-sectional study in the US population and Mendelian randomization analysis in the European population. *Front Immunol.* (2024) 15:1377432. 10.3389/fimmu.2024.1377432 38863716 PMC11165030

[18] March L Schwarz J Carfrae B Bagge E. Clinical validation of self-reported osteoarthritis. *Osteoarthritis Cartilage.* (1998) 6:87–93. 10.1053/joca.1997.0098 9692063

[19] Gould D Dowsey M Jo I Choong P. Patient-related risk factors for unplanned 30-day readmission following total knee arthroplasty: A narrative literature review. *ANZ J Surg.* (2020) 90:1253–8. 10.1111/ans.15695 31970878

[20] Schmidt M. Cardiovascular risks associated with non-aspirin non-steroidal anti-inflammatory drug use. *Dan Med J.* (2015) 62:B4987. 25909098

[21] Hall A Stubbs B Mamas M Myint P Smith T. Association between osteoarthritis and cardiovascular disease: Systematic review and meta-analysis. *Eur J Prev Cardiol.* (2015) 23:938–46. 10.1177/2047487315610663 26464295

[22] Motta F Barone E Sica A Selmi C. Inflammaging and osteoarthritis. *Clin Rev Allergy Immunol.* (2022) 64:222–38. 10.1007/s12016-022-08941-1 35716253

[23] Knights A Redding S Maerz T. Inflammation in osteoarthritis: The latest progress and ongoing challenges. *Curr Opin Rheumatol.* (2023) 35:128–34. 10.1097/BOR.0000000000000923 36695054 PMC10821795

[24] Sammartino A Falco R Drera A Dondi F Bellini P Bertagna F Vascular inflammation and cardiovascular disease: Review about the role of PET imaging. *Int J Cardiovasc Imaging.* (2022) 39:433–40. 10.1007/s10554-022-02730-9 36255543 PMC9870832

[25] Ma T Wang X Qu W Yang L Jing C Zhu B Osthole suppresses knee osteoarthritis development by enhancing autophagy activated via the AMPK/ULK1 pathway. *Molecules.* (2022) 27:8624. 10.3390/molecules27238624 36500713 PMC9738845

[26] Liberale L Badimon L Montecucco F Lüscher T Libby P Camici G. Inflammation, aging, and cardiovascular disease: JACC review topic of the week. *J Am Coll Cardiol.* (2022) 79:837–47. 10.1016/j.jacc.2021.12.017 35210039 PMC8881676

[27] Corsi M Alvarez C Callahan L Cleveland R Golightly Y Jordan J Contributions of symptomatic osteoarthritis and physical function to incident cardiovascular disease. *BMC Musculoskelet Disord.* (2018) 19:393. 10.1186/s12891-018-2311-4 30414614 PMC6230250

[28] Anderson J Felson D. Factors associated with osteoarthritis of the knee in the first national Health and Nutrition Examination Survey (HANES I). Evidence for an association with overweight, race, and physical demands of work. *Am J Epidemiol.* (1988) 128:179–89. 10.1093/oxfordjournals.aje.a114939 3381825

[29] Teichtahl A Wluka A Tanamas S Wang Y Strauss B Proietto J Weight change and change in tibial cartilage volume and symptoms in obese adults. *Ann Rheum Dis.* (2015) 74:1024–9. 10.1136/annrheumdis-2013-204488 24519241

[30] Goulston L Kiran A Javaid M Soni A White K Hart D Does obesity predict knee pain over fourteen years in women, independently of radiographic changes? *Arthritis Care Res.* (2011) 63:1398–406. 10.1002/acr.20546 21739621

[31] Restuccia R Ruggieri D Magaudda L Talotta R. The preventive and therapeutic role of physical activity in knee osteoarthritis. *Reumatismo.* (2022) 74:1–21. 10.4081/reumatismo.2022.1466 35506320

[32] Chang A Lee J Chmiel J Almagor O Song J Sharma L. Association of long-term strenuous physical activity and extensive sitting with incident radiographic knee osteoarthritis. *JAMA Netw Open.* (2020) 3:e204049. 10.1001/jamanetworkopen.2020.4049 32364594 PMC7199114

[33] Liu J Jia S Yang Y Piao L Wang Z Jin Z Exercise induced meteorin-like protects chondrocytes against inflammation and pyroptosis in osteoarthritis by inhibiting PI3K/Akt/NF-κB and NLRP3/caspase-1/GSDMD signaling. *Biomed Pharmacother.* (2023) 158:114118. 10.1016/j.biopha.2022.114118 36527845

[34] Rotter Sopasakis V Wickelgren R Sukonina V Brantsing C Svala E Hansson E Elevated glucose levels preserve glucose uptake, hyaluronan production, and low glutamate release following interleukin-1β stimulation of differentiated chondrocytes. *Cartilage.* (2018) 10:491–503. 10.1177/1947603518770256 29701083 PMC6755873

[35] Eitner A Culvenor A Wirth W Schaible H Eckstein F. Impact of diabetes mellitus on knee osteoarthritis pain and physical and mental status: Data from the osteoarthritis initiative. *Arthritis Care Res.* (2021) 73:540–8. 10.1002/acr.24173 32105401

[36] Li Q Wen Y Wang L Chen B Chen J Wang H Hyperglycemia-induced accumulation of advanced glycosylation end products in fibroblast-like synoviocytes promotes knee osteoarthritis. *Exp Mol Med.* (2021) 53:1735–47. 10.1038/s12276-021-00697-6 34759325 PMC8639977

[37] Peshkova M Lychagin A Lipina M Di Matteo B Anzillotti G Ronzoni F Gender-related aspects in osteoarthritis development and progression: A review. *Int J Mol Sci.* (2022) 23:2767. 10.3390/ijms23052767 35269906 PMC8911252

[38] Nedunchezhiyan U Varughese I Sun A Wu X Crawford R Prasadam I. Obesity, inflammation, and immune system in osteoarthritis. *Front Immunol.* (2022) 13:907750. 10.3389/fimmu.2022.907750 35860250 PMC9289681

[39] Zhang Y Wang J Liu X. Association between hypertension and risk of knee osteoarthritis: A meta-analysis of observational studies. *Medicine.* (2017) 96:e7584. 10.1097/MD.0000000000007584 28796041 PMC5556207

